# Toxicity Mechanisms of Copper Nanoparticles and Copper Surfaces on Bacterial Cells and Viruses

**DOI:** 10.3390/ijms241310503

**Published:** 2023-06-22

**Authors:** Javiera Ramos-Zúñiga, Nicolás Bruna, José M. Pérez-Donoso

**Affiliations:** BioNanotechnology and Microbiology Laboratory, Center for Bioinformatics and Integrative Biology (CBIB), Faculty of Life Sciences, Universidad Andres Bello, Santiago 8370186, Chile; ramoszjaviera@gmail.com (J.R.-Z.); n.brunarivera@gmail.com (N.B.)

**Keywords:** antimicrobial copper, copper toxicity, copper mechanisms, antimicrobial agent, antiviral agent, copper nanoparticles

## Abstract

Copper is a metal historically used to prevent infections. One of the most relevant challenges in modern society are infectious disease outbreaks, where copper-based technologies can play a significant role. Currently, copper nanoparticles and surfaces are the most common antimicrobial copper-based technologies. Despite the widespread use of copper on nanoparticles and surfaces, the toxicity mechanism(s) explaining their unique antimicrobial properties are not entirely known. In general, toxicity effects described in bacteria and fungi involve the rupture of membranes, accumulation of ions inside the cell, protein inactivation, and DNA damage. A few studies have associated Cu-toxicity with ROS production and genetic material degradation in viruses. Therefore, understanding the mechanisms of the toxicity of copper nanoparticles and surfaces will contribute to developing and implementing efficient antimicrobial technologies to combat old and new infectious agents that can lead to disease outbreaks such as COVID-19. This review summarizes the current knowledge regarding the microbial toxicity of copper nanoparticles and surfaces and the gaps in this knowledge. In addition, we discuss potential applications derived from discovering new elements of copper toxicity, such as using different molecules or modifications to potentiate toxicity or antimicrobial specificity.

## 1. Copper Properties and Applications

Copper is a heavy metal with three oxidation states: metallic copper, cuprous ion, and cupric ion; the last two being stable [1]. This element is essential for life in all prokaryotic and eukaryotic organisms due to its structural and functional role as an enzymatic cofactor of proteins involved in metabolic pathways such as cell respiration, photosynthesis, ion transport, hormone production, growth, and cell development [2,3,4,5]. Cellular requirements of this metal vary depending on the species, environment, and metabolic state [5]. Despite being an essential element, high concentrations of copper are toxic for most organisms. Copper toxicity is mainly associated with the ability to generate hydroxyl radicals (ROS) through the Fenton and Haber–Weiss reactions, triggering damage to lipids, proteins, and DNA and affecting cell viability [4,6].

Copper is widely used worldwide due to its versatility and antimicrobial activity. Copper-based technologies are currently applied in construction, textiles, agriculture, and health, among others (Table 1). For example, copper ions are used as a photoluminescence activator due to their conductive capacity [7]. In agriculture, solutions based on copper compounds are used in fruit and vegetable crops to prevent bacterial and fungal infections [8,9]. In the livestock industry, copper has been used as a diet supplement to promote pigs’ growth and prevent bacterial diseases such as *Escherichia coli*-mediated mastitis [9,10,11]. The use of copper in hospital settings to prevent healthcare-associated infections (HAIs) by *E. coli*, methicillin-resistant *Staphylococcus aureus* (MRSA), *Listeria monocytogenes*, *Clostridium difficile*, and influenza A virus, among many other microorganisms, has been reported [12,13,14,15,16,17]. Copper has also been used as nanoparticles (NPs): Rai et al. (2018) discussed the different uses of copper nanoparticles for food preservation. It has been reported that using bimetal nanoparticles composed of Ag-NPs and Cu-NPs added to an agar film inhibits the growth of microorganisms in food packaging [2,3,4]. The use of copper and Cu-NPs in water treatment processes has also been described where copper solutions are used for their antimicrobial activity in water and copper-based nanoparticles as an adsorbent material [18,19].

This review focuses on copper as an antimicrobial and antiviral agent, with special interest in the toxicity mechanism of copper nanoparticles and surfaces. Due to the widespread application and investigation of copper in recent years, it is essential to deeply understand how this metal works in terms of their toxicity mechanisms and its effects on cells. This knowledge will contribute to potentiate the capabilities of this material as an antimicrobial. Based on this, the terms used to search and select the literature were copper toxicity, antimicrobial copper, antiviral copper, copper nanoparticles, copper nanoparticle toxicity mechanisms, copper surface mechanisms, copper surfaces, and contact killing. Based on these criteria, literature articles that discussed or proposed mechanisms of toxicity and articles reporting copper-based clinical applications were included in this review.

**Table 1:** Copper-based technologies.

Application	References
Agriculture and Livestock	
Fungicide, algicide, nutritional supplement	[8,9,20,21,22]
Building	
Electricity, wiring, plumbing, cooling, roofing	[1,9]
Industrial	
Water treatment, dye manufacturing, oil refining, wood preservation	[8,9,23,24]
Health	
Intrauterine devices, dental crowns, antimicrobial surfaces, face masks	[12,25,26,27]
Others	
Photoluminescence	[7]
Textile	[25,28]
Food Industry	[9]
Carbon–copper Nanotubes	[29]
Transformation of solar energy, batteries, and gas sensors	[30,31]

## 2. Use of Copper as an Antimicrobial

Copper has been used as an antimicrobial agent throughout history. In ancient Egypt, copper was used for medical purposes to disinfect wounds and drinking water [32,33]. Subsequently, other civilizations adopted this idea; Greeks, Romans, and Aztecs used copper and derived compounds for treating burns, intestinal worms, and infections [32,34]. The use of this metal extended until the commercial development of antibiotics in 1945 [35]. However, because of the spread of antibiotic-resistant microorganisms, during the last few decades, the use of copper as an alternative to dealing with resistant microorganisms has emerged as a powerful strategy [35,36]. Copper is a flexible material used in different formats, such as on surfaces, as nanoparticles, and in solutions, to prevent the spread of pathogens. It is durable over time and does not require continuous maintenance or treatment to be effective, making it a long-term investment. Moreover, the constant use of copper has been considered safe for humans, as in the case of intrauterine devices, copper pipes, and dental amalgam alloys [37]. Studies of copper textiles showed no skin irritation or adverse reactions in animals. In 2008, the US Environmental Protection Agency (EPA) declared copper and all its alloys as the first effective metallic antimicrobial agent, spreading its application in different industries [36]. Currently, various industries use copper’s antimicrobial properties in two forms: solid (surfaces and nanoparticles) and ionic (salts). Table 2 describes some of the main applications of copper as an antimicrobial. The advantages of using copper as an antimicrobial agent are associated with the low dose required to eliminate the microorganisms and the infrequent evolution of resistance mechanisms [27].

In addition, copper can act effectively against persistent and dormant cells [24,38]. Compared to antibiotics, this metal damages cells by affecting multiple processes, including membrane damage, genotoxicity, ROS production, protein dysfunction, and nutrient assimilation [24].

The main objective of using copper on surfaces is to prevent the spread of infections from contaminated surfaces. This type of material, with optimal surface disinfection, reduces the presence of microorganisms [39]. Copper surfaces are highly effective in preventing the formation of bacterial biofilms. For example, their use in the poultry industry to prevent biofilm formation by *Salmonella enteritidis* has been recently reported [40]. Copper surfaces also prevent the transfer of genes involved in antibiotic resistance in different organisms because they eradicate the genetic material [39]. In this way, it is possible to prevent the horizontal transfer of antibiotic-resistance genes between microorganisms on surfaces [41]. Using copper as an antimicrobial is a promising alternative to fight antibiotic-resistant microorganisms such as methicillin-resistant *Staphylococcus aureus* (MRSA) or viruses such as SARS-CoV-2. Copper inactivates a broad spectrum of microorganisms such as bacteria, fungi, viral, and spores [42], and most clinical microorganisms present low copper-resistance levels [43]. Unlike antibiotics, copper affects various cellular or viral targets, causing membrane and capsid destabilization, ROS production, protein damage, enzyme inhibition, displacement of Fe–S centers, and genotoxicity [3,4,5,42,44,45,46]. In recent years, the use of copper in medical devices has increased, with 30% of the articles cited in Table 2 reporting the use of copper surfaces in hospitals or clinical scenarios. Copper surfaces reduce the microbial load and HAI infection rates within health facilities [47,48,49]. In turn, the use of copper has positive effects when adhered to titanium surfaces and in Ti/Cu alloys used in medical implants [50,51]. Although the price of pure copper is higher than other metals or alloys used in clinical settings, such as aluminum or bronze, which are commonly used in medical equipment, copper is a less-expensive metal than titanium, gold, silver, or stainless steel, which are also widely used in the clinical field [52]. The scientific evidence provided in this review indicates that copper has antimicrobial properties that may favor its application in hospitals or public spaces. However, more robust scientific evidence still needs to be developed to accelerate the widespread use of copper in these settings.

The use of copper-based nanoparticles (Cu-NPs)—CuO, Cu_2_O, Cu^0^, CuS, and CuI—with antimicrobial properties is also important because of the intrinsic characteristics of these materials at the nanometric scale: their surface area, mechanical, chemical, electrical, optical, and magnetic characteristics are different from the ionic form [53]. Some of the uses of copper as an antimicrobial in nanotechnology applications involve the incorporation of Cu-NPs in cotton fibers for medical dressings and impregnated supplies [25,54]. These are also used in the textile industry, for example, socks with Cu-NPs are manufactured to prevent infections by “athlete’s foot” fungus and diabetic foot [25,37]. During the last few years, the applications of Cu-NPs for preventing the spread of HAIs have increased, and they have been implemented in different hospital environments. Table 2 provides several articles describing the use of copper, where more than 50% of the articles correspond to clinical applications of copper-based NPs. These NPs have also been used in anti-folding catheters to prevent the formation of biofilms by pathogens or multi-resistant organisms [55,56,57], prostheses [58,59,60,61], air filters [34,57,58], composite films with chitosan and copper oxide nanoparticles, and starch hydrogels with copper nanoparticles for wound-dressing applications [62,63]. Furthermore, copper-based NPs have been used in dentistry in long-lasting adhesive restoration materials [64], adhesive resins [65,66], and implant coatings [67]. In addition, Cu-NPs incorporated into the cover and paints of waiting room chairs have contributed to reducing the bacterial load in hospitals [68]. Cu-NPs are also used in respiratory face masks against human influenza A (H1N1) [25]. This application is relevant in the context of the current pandemic to prevent the spread of the severe acute respiratory syndrome coronavirus 2 (SARS-CoV-2). Recently, in 2021, the EPA registered copper surfaces for their use against coronaviruses (Environmental Protection Agency, 2021). As a result of the current situation, many studies of copper as an antiviral to prevent the spread of SARS-CoV-2 have been implemented. Among them, the use of copper surfaces in combination with nanoparticles to generate a synergistic effect against viruses has been recently reported [69,70]. Technologies based on Cu-NPs or copper solutions have also been used to improve the composition of personal protective equipment (PPE) [49,71,72].

**Table 2:** Examples of copper application as an antimicrobial.

Application	Function	Type of Copper	References
Copper surfaces	Norovirus inactivation	Cu-alloy surfaces	[73]
Copper surfaces	Elimination of vancomycin-resistant *Enterococci*	Cu^0^ and Cu-alloy surfaces	[39,74]
Antimicrobial surfaces in spaceflight	Prevention of *S. cohnii* and *E. coli* infections	Cu^0^ and CuO/Cu_2_O	[75]
Surfaces in pediatric intensive care unit	Prevention of infections associated with hospitals	Cu^0^ surfaces	[55,76]
Copper-doped silica xerogels	Avoidance of skin infections	Cu ions	[56]
Wound dressings	Prevention of *S. aureus* and *K. pneumoniae* infections	CuO/Cu_2_O NPs	[25]
Application of nanotechnology in textiles, latex gloves, and polymers	Prevention of microorganisms in medical supplies	CuO/Cu_2_O NPs	[8,25,30]
Antiviral filters	Dialysis pumps, blood banks and air filters	CuO/Cu_2_O NPs	[34,57,58]
Cotton fibers	Prevention of bacteria, fungi and viruses	Cu ions and NPs	[54]
Surfaces with nanoparticle inlay	Antimicrobial activity	Cu^0^ NPs	[31,59]
Office, kindergarten, retirement home, hospital facilities	Antimicrobial activity	Cu/brass surfaces	[60]
Face masks	Antiviral activity against influenza A	CuO/Cu_2_O NPs	[25]
Dental materials	Bacterial plaque prevention	CuO/Cu_2_O NPs	[64,65,66,67]
Prostheses	Antibacterial 3D filaments	Cu-NPs	[77]
Polyester surface-coating paint	Antimicrobial activity	Cu-NPs	[68]
Wound dressing	Antibacterial activity	Bacterial celullose/Cu-NPs	[78]
Nanostructures	Antimicrobial– Antibiofilm activity	Cu-NPs/Carbon nanotubes	[79]
3D face masks, connector for ventilators	Antiviral activity against SARS-CoV-2	Cu-NPs	[80]
Antibacterial nanomaterials	Multidrug-resistant bacterial treatment	CuS NPs/ Graphene oxide nanosheets	[81]
Antibacterial coatings	Antibacterial activity Antibiofilm activity	CuS nanoparticles	[82]
Poultry industry	Antibiofilm formation by *Salmonella enteritidis*	Cu surface	[40]
Nanostructured copper surface/Prototypes for air and water cleaning	Antiviral activity against SARS-CoV-2	Copper surface and copper nanoparticles	[70]
Transparent surface coating	Antiviral activity against Influenza A	Cu-NPs– Graphene	[71]
Spray coating	Antiviral activity against SARS-CoV-2	Cu-NPs	[72]
Nanocomposite surfaces	Inhibition of SARS-CoV-2 and HuNV viruses	Cu–Ag nanocomposite surfaces	[69]
Textile coating	SARS-CoV-2 inactivation	Hybrid alginate–copper sulfate	[49]
Photothermal treatment	Inhibition of bacteria in infected skin	CuS NPs	[83]

## 3. Copper Toxicity Mechanisms

### 3.1. Copper-Based Nanoparticles

**Figure 1:**
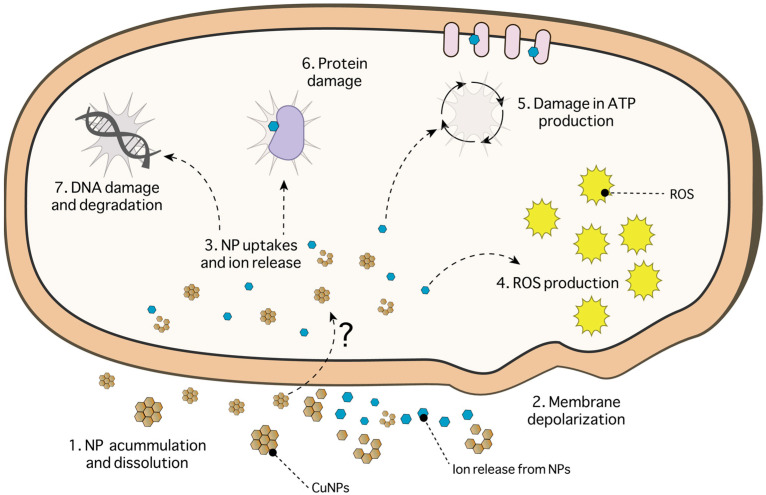
General mechanism of toxicity for copper-based nanoparticles. (1) Accumulation, dissolution, and entry of nanoparticles into the cell. (2) Depolarization and membrane rupture due to the accumulation of nanoparticles on the cell surface [24,107,108,109]. (3) Cell entry of the nanoparticles and release of Cu (II) ions because of NP destabilization. The release of ions causes the following effects: (4) ROS production, lipid peroxidation, and protein oxidation; (5) Decrease in ATP production; (6) Protein damage due to oxidation and iron replacement by copper in proteins with Fe–S centers; and (7) DNA damage and degradation [84,98,99,108,110].

Interest in copper-based nanoparticles (Cu-NPs) has grown over the last few years because of their antimicrobial properties against bacteria, fungi, viruses, and algae [84]. To date, different Cu-NPs with antimicrobial properties have been described, including CuO/Cu_2_O, CuS, and Cu^0^ NPs. Concerning the former, Giannousi et al. (2014) described the degradation of plasmid DNA in bacterial strains using CuO/Cu_2_O NPs (Figure 1) [85]. This degradation occurs depending on the dose of nanoparticles used. Furthermore, they showed that the concentration of ions released was below that needed for the inhibition of bacterial growth (Minimum Inhibitory Concentration). Other authors have evaluated the antimicrobial activity of copper oxide nanoparticles and their size effect. Applerot et al. (2012) and Padil et al. (2013) reported that copper oxide nanoparticles of small size (~5 and ~4.8 nm, respectively) had better antimicrobial activity than nanoparticles of larger size (~7.8 nm) [30,86]. This phenomenon occurs due to the capacity of smaller nanoparticles to penetrate the cells (Figure 1) [87,88,89,90].

On the other hand, the antimicrobial effect of CuS nanoparticles has been scarcely reported, even though these NPs are much less toxic for microorganisms than copper oxide or Cu^0^ nanoparticles. ROS production mediated by the Cu ions released from the nanostructure is the main cause of the cellular effects of CuS NPs (Figure 1) [91,92]. However, the antimicrobial effect of these nanoparticles could also be due to their nanometric size, composition, and structure [93,94,95].

Regarding Cu^0^ NPs, several articles have contributed to elucidating their antimicrobial mechanism (Figure 1). The toxicity mechanism of these NPs involves the interaction, accumulation, and dissolution of the NPs in the membrane, changing their permeability. Consequently, the proton motive force of the membrane decreases [96]. The generation of ROS by ions released from the nanoparticles causes lipid peroxidation, protein oxidation, and DNA degradation [96,97]. In turn, the metal ions inside the cell damage the DNA and affect ATP production. Copper ions can interact with phosphate and -SH groups of proteins and DNA, causing denaturation and other structural perturbations [84,96,98,99]. These antecedents suggest that the toxicity of Cu^0^ NPs is mediated by the release of Cu^+2^ ions when metal nanoparticles are oxidized.

In general, it has been described that the toxicity of Cu-NPs is associated with the adhesion of the NPs to the cell through electrostatic interactions [100,101,102]. As mentioned above, adhesion to the membrane could involve depolarization and cell death (Figure 1) [96,103]. The interaction of Cu-NPs with the cell membrane and the formation of holes in the membrane that allow the entry of Cu-NPs inside cells (Figure 1) has been described [104]. However, despite the evaluated background, the specific toxicity mechanism for each type of copper NPs has not been fully explained.

The toxicity of Cu-NPs depends on many factors such as temperature, pH, concentration, number of bacterial cells, and intrinsic physicochemical properties of the nanostructures, such as shape, size, and surface composition [53,103,105]. The interaction of small-sized nanoparticles and plasma membranes has been described; this interaction could generate a large contact surface between the metal ions and the cell, generating cell damage [30,86,93,94,94,95]. Shape and surface charge also influence the cell contact area, thus increasing its efficiency [95,106]. This background leaves a window for future research that may contribute to elucidating the mechanism of toxicity of CuO, Cu-S, and Cu^0^ nanoparticles.

### 3.2. Copper Surfaces

The toxicity mechanisms of metallic copper surfaces and copper alloys are not totally known. However, contact killing has been proposed as the main mechanism involved in the elimination of microorganisms present on surfaces [36]. Contact killing is a mechanism that explains the cellular damage associated with the direct contact of a microorganism with surfaces composed of metals, surfaces coated with biocidal substances, or antimicrobial substances [35,45,46,111,112]. This mechanism of toxicity involves direct physical interaction with the organism, has immediate action and long-term effects, and does not require repeated applications for more effectiveness.

**Figure 2:**
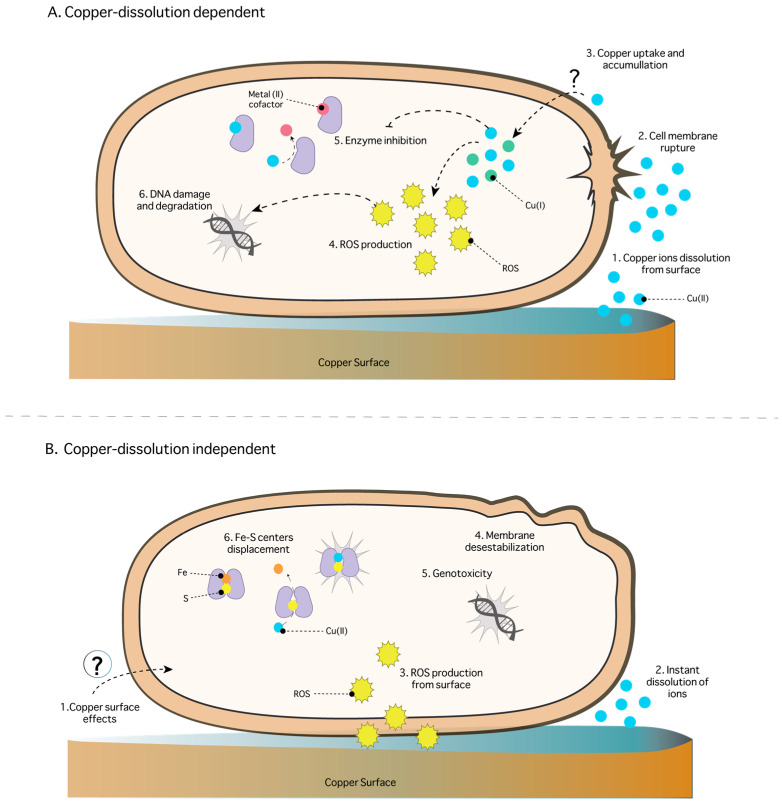
General mechanisms of contact killing on surfaces. (**A**) The “wet” method depends on copper dissolution: (1) Dissolution of copper ions from the metal surface [112]. (2) Cell membrane rupture due to the accumulation of ions in the membrane. (3) Copper entry and accumulation inside the cell. (4) ROS production due to the accumulation of ions within the cell. (5) Inhibition of essential enzymes by competition for divalent cation-binding sites used as cofactors [44]. (6) Damage and degradation of genomic and plasmid DNA because of the accumulation of ions and free radicals [3,5,6,32,45,112]. (**B**) “Dry” method independent of copper dissolution: (1) The toxic effect of the dry surface without ion release is unknown. Regarding this, (2) the effect of the surface due to the quick and concentrated dissolution of ions during the inoculation is postulated [39]. (3) ROS production by the accumulation of ions. (4) Membrane destabilization [42], (5) genotoxicity, and (6) displacement of Fe–S centers in proteins [44].

In general, two methods have been regularly used to evaluate the toxicity of copper surfaces: “wet” and “dry” [5,111]. The “wet” application refers to the inoculation technique, where a suspension of bacteria is added to the copper coupons (Figure 2A). The “dry” method uses a small amount of liquid spread through a cotton cane (Figure 2B). The “wet” mechanism begins with the dissolution of copper ions from the surface, generating cellular damage resulting from the entry of copper ions [32,36,45,112]. As shown in Figure 2A, the contact-killing mechanism involves damage to the plasma membrane due to the stress caused by the dissolution of copper ions from the surface, the admission and accumulation of high concentrations of Cu^+1^ and Cu^+2^ inside the cell [3,6,32], and the intracellular production of hydroxyl radicals as a result of Fenton-like reactions between Cu^+1^ and Cu^+2^ in the presence of H_2_O_2_ [5,6,32,46]. Copper ions released from the surface enter the cell and compete for metal-binding sites in enzymes (Figure 2A), generating the inhibition of essential enzymes and the destruction of iron–sulfur centers. In this way, copper displaces iron by coupling to sulfur and inactivating enzymes [5,6,32,44]. Finally, the DNA structure is broken or degraded due to the ROS generated in the cell (Figure 2A) [5,32,36,113].

The effect of copper surfaces on bacterial respiration has also been reported on vancomycin-resistant *Enterococcus* species in a process associated with cytochrome inhibition by copper ions released from the surface [39]. Warnes et al. (2012) reported that in *E. coli* O157: H7 and *Salmonella*, the damage caused by hydroxyl radicals is not the primary toxicity mechanism of copper surfaces [114]. The authors mainly refer to the damage occurring on the outer membrane since it is the cellular structure that interacts the most with the surface. Concerning this hypothesis, some studies demonstrate the high affinity of lipopolysaccharides with divalent cations in the extracellular space [115]. This phenomenon could influence the ion concentration in the membrane and its consequent rupture.

On the other hand, in the “dry” method, the liquid evaporates immediately, allowing direct contact of the cells with the metal surface [32]. Microorganisms exposed to the dry-surface method present higher sensitivity to copper surfaces. The time of death is shorter in bacteria and yeasts exposed to dry surfaces [116,117,118]. To date, it is still unknown why the “dry” or dissolution-independent method has higher antimicrobial efficacy than the “wet” method, and it seems that the cellular toxicity mediated by dry surfaces could involve an independent mechanism of copper solubilization (Figure 2B). The mechanism involved in the “dry” conditions is unknown. It has been suggested that it could involve ROS production. Warnes et al. (2011) indicate that exposure to copper on “dry” surfaces could involve the accumulation of ions immediately upon contact with the surface. This effect decreases the membrane potential in the cell, causing its destabilization. Santo et al. (2011) determined that “dry” toxicity in *E. coli* causes membrane damage due to ion accumulation, this being the primary mechanism of the previously described genotoxicity [42]. In addition, the inhibitory effect of copper on LD-transpeptidases associated with peptidoglycan has been reported in *E. coli* and *E. faecium* [119]. Using quantitative proteomics, Nandakumar et al. (2011) identified some of the main changes in cells exposed to “dry” conditions. This study supports the hypothesis that the cell envelope is mainly affected by copper surfaces due to the increase in the expression of proteins associated with the shell and the polysaccharide biogenesis of the capsule [119,120]. The overexpression of this machinery could be related to the repair or response to the damage caused by copper. Regarding the genotoxicity associated with copper on surfaces, the presence of DNA-repair proteins has not been reported [41]. In addition, proteins with iron-sulfur centers, such as isopropyl malate dehydratase, have been described as being inactivated in the presence of copper. This phenomenon results from the displacement of iron–sulfur centers [44].

We have previously mentioned some mechanisms involved in the toxicity of copper surfaces. However, most of these processes are exclusively related to the dissolution of ions from the metal surface. In both conditions (wet and dry), microorganisms cannot grow on the surface and the inactivation time is short: minutes to hours in the case of “wet” conditions and minutes in “dry” conditions. Finally, the key steps that influence contact killing in microorganisms are the dissolution of copper on surfaces, the bacteria–metal contact, the surface structure, and the nobility of the metal alloys [42,45,112,121,122,123]. Among these, bacteria–metal contact is the most critical step. Mathews et al. demonstrated the role of the physical contact of bacteria with the metal surface by blocking the copper surface with an inert polymer. Their study showed a decrease in the toxicity of copper surfaces in the absence of direct contact [122].

Considering the current knowledge regarding copper surface toxicity and their increasing applications that impact millions of humans daily in public environments, it is necessary to understand the effect of copper surfaces on microbial communities, to determine the microorganisms most capable of surviving on them, and to elucidate if these surfaces generate a selection pressure favoring microorganisms of health interest. Santo et al. (2010) isolated microorganisms present in European copper-composite currencies [117]. The authors determined that Gram-positive bacteria are more predominant in these samples. It would be ideal to evaluate the population associated with these surfaces by genomic-based analysis to determine the influence of copper on microbial communities.

## 4. Antiviral Effect of Copper Nanoparticles and Surfaces

Viruses are highly susceptible to copper-mediated toxicity because viruses lack a metal response or repair mechanisms such as those that exist in microorganisms [34,58]. Table 3 summarizes studies on copper toxicity on surfaces, as nanoparticles, and in solutions, its effect on different viruses (enveloped or non-enveloped), and the associated mechanism. To date, no correlation between copper susceptibility and virus type has been determined. Virus envelope characteristics, family, and genetic material are not directly related to copper toxicity [57].

### 4.1. Copper Nanoparticles as an Antiviral Agent

Many articles evaluating the effect of different Cu-NPs—CuO, Cu_2_O, Cu^0^, CuS, and CuI—against viruses have been reported to date. Table 3 summarizes some examples of the effect of these Cu-NPs on enveloped and non-enveloped viruses. In this section of the review, we will focus on the mechanisms described for each type of Cu-NP.

To date, most reports describing the use of Cu-NPs as antiviral agents study the effect of copper oxide NPs (CuO/Cu_2_O) over coated and uncoated viruses. Tavakoli and Hashemzadeh (2020) described that the mechanisms associated with the antiviral activity of CuO nanoparticles against Herpes Simplex Virus (HSV-1) involve the interaction with the virus surface (Table 3). In this case, nanoparticles affect viral attachment and cell entry [124]. The same process occurs when copper oxide nanoparticles interact with the Hepatitis C virus (HCVpp), blocking its entry into the cells [125]. In other viruses such as respiratory syncytial virus (RSV), Cytomegalovirus (CMV), Adenovirus Type I (HAdV), and Rhinovirus 2 (HRV-2), CuO NPs cause a reduction in viral titers [57].

Antiviral activity has also been reported in CuS NPs (Table 3). In the HuNoV virus, CuS NPs could disrupt capsid proteins, causing protein degradation [126]. Recently, Guo et al. (2021) described the use of CuS NPs to prevent Hepatitis B virus infections (HBV) [127]. The authors synthesized ultra-small Cu_2_S nanoparticles that blocked HBV assembly after photoinduction. Their study showed that the size of the nanoparticles also influences the antiviral effect. Further studies will be necessary to evaluate future applications of this type of nanomaterials as an antiviral.

On the other hand, Fujimori et al. (2012) determined that the antiviral mechanism of copper(i) iodide (CuI) particles against influenza H5N1 virus involves the generation of hydroxyl radicals. This radical could degrade functional viral proteins, such as hemagglutinin (HA) and neuraminidase (NA). Furthermore, the oxidation of lipids from the virus coating was also reported [128].

**Figure 3:**
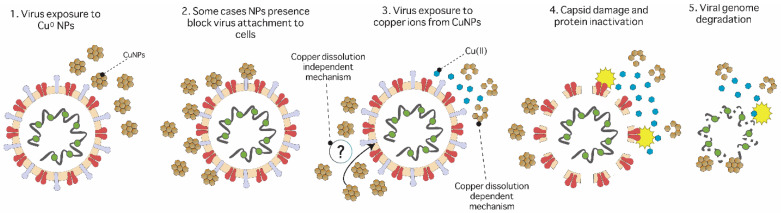
General toxicity mechanisms of copper-based nanoparticles against viruses. (1) The virus is exposed to copper-based NPs. (2) In some cases, NPs could block the virus attachment to cells by blocking or inactivating proteins involved in this process [125]. (3) The virus interacts with copper-based NPs; this mechanism is unknown, but it is suggested that NPs could destabilize the capsid membrane (left). Conversely, copper-based NPs release copper ions (right). In both cases, the virus is exposed to a high concentration of localized copper, which causes (4) damage to the capsid, inactivation of proteins, and (5) degradation of the genetic material [126].

In general, the toxicity mechanisms of copper-based NPs on viruses have been associated with damage to the viral genome, inhibition of replication, protein synthesis, and the assembly and release of virions [124,125,126,128,129]. Figure 3 shows the main effects of Cu-NPs on viruses. There are two possible mechanisms explaining toxicity of NPs on viruses. One is an unknown dissolution-independent mechanism where the NPs remain stable and “accumulate” high concentrations of copper that can “attack” the virus. The second mechanism involves NP destabilization, where high concentrations of copper ions are generated. These generate capsid degradation, protein inactivation, and viral genome damage [126]. The common factor described in all Cu-NPs corresponds to “virus neutralization” in the case of RSV, CMV, HadV, and HRV-2 [57]. Specifically, it has been reported that Cu-NPs can block the attachment and cell entry of the HCV virus [125]. In the HSV-1 and HuNoV viruses, Cu-NPs can inactivate viral proteins via oxidation and degradation of the viral genome (Figure 3). It will be essential to consider these antecedents to optimize the use of copper NPs as antiviral agents in future applications.

### 4.2. Antiviral Effect of Copper Surfaces

**Figure 4:**
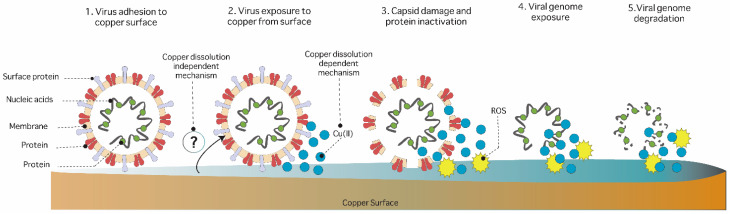
Toxicity mechanisms of copper surfaces against viruses. (1) The viral particle adheres to the copper surface, and (2) the virus is exposed to the copper surface. Two mechanisms have been proposed. Left: the virus is damaged by the surface independently of copper ion release; this mechanism is unknown. Right: the virus is damaged by copper ions released by the metal surface. (3) Exposure to copper ions causes capsid damage and protein inactivation associated with ROS. (4–5) Genetic material is exposed and degraded [73,131,133].

In viruses, the toxicity mechanism is not associated with metabolic processes. In this regard, surface mechanisms could be associated with virus immobilization and genetic material inactivation. It has also been related to blocking or destroying the viral receptors for host cells [130]. The antiviral mechanism of copper surfaces has been associated with the destruction of viral genetic material and damage to the capsid surface, which could be disintegrated by the effect of copper ions released from the metal surface (Figure 4) [36,73].

The mechanism of virus inactivation on copper surfaces is shown in Figure 4. First, this process involves surface adhesion, such as that previously described in bacteria. The toxicity of copper surfaces against viruses could be a dependent or independent effect of the dissolution of copper ions from the surface. The dependent mechanism is associated with capsid damage and the degradation of proteins and DNA produced by the release of high concentrations of copper ions from the surface [73,131]. Table 3 summarizes different antiviral applications of copper surfaces on enveloped and non-enveloped viruses and the mechanisms described for these. RNA damage was reported when the influenza A virus was exposed to copper surfaces [15]. It has been reported that copper exposition involves capsid damage, loss of spike proteins, and viral genome degradation [132,133]. RNA degradation and capsid damage was observed when the non-enveloped virus human norovirus (HuNoV) was exposed to dry surfaces and copper alloys [73,131] The mechanism of toxicity of copper surfaces is independent of the virus type, whether enveloped or non-enveloped. However, most studies are associated with enveloped viruses such as Influenza A, Human coronavirus 229E, and SARS-CoV-2 [15,73].

**Table 3:** Studies of copper as an antiviral agent and the toxicity mechanisms proposed.

Virus	Disease	Nucleic Acid Type	Family	Copper Type	Proposed Mechanism	References
Enveloped						
Influenza A	Respiratory tract infection	Single-stranded RNA	*Orthomyxoviridae*	Cu surface	RNA damage	[15]
				CuI NPs	Viral protein degradation	[128]
Hepatitis C virus (HCV)	Hepatic cirrhosis and hepatocellular carcinomaoma	Single-stranded RNA	*Flaviviridae*	CuO NPs	Blockage of attachment and entry stages	[125]
Hepatitis B virus (HBV)	Acute and chronic viral hepatitis	Double-stranded DNA	*Hepadnaviridae*	Cu_2_S NPs		[47]
Herpes Simplex Virus (HSV-1)	Labial lesions, encephalitis, and peripheral nervous system disorders	Double-stranded DNA	*Herpesviridae*	CuO NPs	Inactivation via oxidation of viral proteins, degradation of viral genome	[124]
Human Immunodeficiency Virus (HIV)	Acquired immunodeficiency syndrome	Single-stranded RNA	*Retroviridae*	Cu ions	HIV-1 protease neutralization	[134]
				CuO filters	HIV-1 protease inactivation, nucleic acid denaturation	[58]
				Cupric ions	Nucleic acid degradation	[135]
Respiratory Syncytial Virus (RSV)	Respiratory illness	Single-stranded RNA	*Paramyxoviridae*	CuO NPs	Viral neutralization	[57]
Cytomegalovirus (CMV)	Mononucleosis	Double-stranded DNA	*Herpesviridae*	CuO NPs	Viral neutralization	[57]
Bronchitis Virus	Inflammation of the bronchial tubes	Single-stranded RNA	*Coronaviridae*	Cu ions	Virus inactivation	[136]
Junin Virus	Argentine hemorrhagic fever	Single-stranded RNA	*Arenaviridae*	Copper ions + peroxide	Inactivation by ROS	[135]
Human coronavirus 229E (HuCoV-229E)	Respiratory illness	Single-stranded RNA	*Coronaviridae*	Cu Surfaces and Alloys	Capsid damage, loss of surface spikes and viral genome inactivation	[133]
Non-enveloped						
Human norovirus (HuNoV)	Acute gastroenteritis	Single-stranded RNA	*Caliciviridae*	Au/CuS NPs	Capsid protein degradation, capsid damage	[126]
				Dry Copper Surfaces and Alloys	RNA degradation and capsid damage	[73,131]
Bacteriophages MS2 and F2	-	Single-stranded RNA	*Leviviridae*	Cupric ions	Phage inactivation	[137]
Bacteriophage R17	-	Single-stranded RNA	*Leviviridae*	Cupric ions	Degradation of phage genome	[138]
Bacteriophage Qß	-	Single-stranded RNA	*Leviviridae*	Particles Cu_2_O, CuO and Ag	ROS, leached copper ions and surface contact	[139]
Poliovirus	Poliomyelitis, paralysis	Single-stranded RNA	*Picornaviridae*	Cu ions	Capsid proteins and RNA attack	[140]
				Cu ions	Inactivation of viral RNA	[141]
Adenovirus Type I (HAdV)	Keratoconjunctivitis	Double-stranded DNA	*Adenoviridae*	CuO NPs	Viral neutralization	[57]
Rhinovirus 2 (HRV-2)	Respiratory illness	Single-stranded RNA	*Picornaviridae*	CuO NPs	Viral neutralization	[57]

NPs—nanoparticles; Cu^0^, CuO, Cu_2_O; Copper ions—CuSO_4_, CI—Copper(i) iodide.

### 4.3. Copper and SARS-CoV-2

In the current COVID-19 pandemic caused by the SARS-CoV-2 virus, using copper surfaces and nanoparticles has become an alternative approach to prevent the spread of the virus. SARS-CoV-2 infection is spread by drops exhaled from the nose or mouth of people infected with the virus. These droplets can be in the air or reach commonly used surfaces [142]. The use of copper in surfaces and nanoparticles has been proposed to prevent the spread of the virus. Copper surfaces have been used in hospitals and public spaces [60,143]. Other studies have modified copper surfaces to prevent infection and the spread of the virus by using a surface coating composed of copper particles and polyurethane [144]. Unlike plastic and metal surfaces, copper surfaces can inactivate the virus by 96–99% after 2 h of exposure [144,145,146]. In addition, copper-based nanoparticles have been used in face masks, PPE, and disinfectants [80,147,148,149]. The effect of disinfectants based on copper iodine (CuI) nanoparticles has been evaluated for the disinfection of PPE; however, the impact has been assessed only in liquid solutions [145].

Previously, Warnes et al. (2015) evaluated the effect of copper surfaces on the human coronaviruses causing severe acute respiratory syndrome (SARS) and the Middle East Respiratory Syndrome (MERS). The authors assessed the human coronavirus 229E on five joint-touch surfaces, including copper and copper alloy surfaces. Rapid inactivation of the coronavirus on copper surfaces depends on the composition of the surface. Inactivation was associated with copper ions released from the surface, ROS generation, and viral genome fragmentation [133]. Aboubakr et al. (2020) and Kampf et al. (2020) studied the persistence of coronaviruses over 2 h to 9 days in different environments, commonly touched surfaces, weather conditions, and biocidal agents [150,151]. Specifically, the authors evaluated surfaces such as steel, aluminum, metal, wood, paper, glass, ceramic, and copper and biocidal agents such as ethanol, 2-propanol, sodium hypochlorite, and hydrogen peroxide. The lowest persistence of SARS-CoV-2 occurs on copper, latex, and soft-porosity surfaces [150,151]. In 2020, Van Doremalen et al. determined the stability of SARS-CoV-1 and SARS-CoV-2 on surfaces and aerosols. Five environmental conditions were assessed (aerosols, stainless steel, plastic, copper, and cardboard), showing that SARS-CoV-2 was not viable after 4 h of copper exposure. The half-life on all surfaces studied was similar for both viruses [132]. The mechanism associated with the toxicity of copper surfaces or nanoparticles against viruses has not been fully defined. Based on the knowledge described above for other viruses, it is most likely that the production of ROS inactivates the viral genetic material.

## 5. Concluding Remarks

As has been seen throughout this review, the use of copper surfaces and nanomaterials as antimicrobials has strongly increased during the last few decades. Since these metallic materials are potential tools for combating future microbial agents, it is crucial to thoroughly explore the pathways involved in their toxicity. The toxic effects of copper are mainly associated with the dissolution of ions from the surface or the destabilization of nanoparticles. These ions cause the rupture and depolarization of the plasma membrane, the production of ROS, the inhibition of essential metabolic enzymes, genotoxicity, and cell death. Considering the literature, there are still many unanswered questions associated with the copper toxicity mechanism, a situation that represents an enormous challenge for the scientific community.

Regarding limitations, Bisht et al. (2022) suggest that one of the main problems limiting the development of new copper applications is that most trials are conducted using in vitro studies [152]; however, it is essential to validate copper applications in field studies before implementing these antimicrobial materials on a large scale. Many factors influence the costs of using copper on a large scale, such as manufacturing, durability, cleaning methods, obtaining certifications, surface maintenance, treatment or coatings, material, and replacement costs. Moreover, through the cleaning methods, copper may lose its aesthetic properties due to metal reactivity, and it is crucial to evaluate if cleaning methods affect its antimicrobial properties [153]. In this sense, it will be helpful for future studies to focus on the efficacy of applying these materials to enhance such properties by finding appropriate cleaning methods and complementing them with coating technologies, and even enhancing synergistic approaches to develop practical and durable surfaces that allow their application in hospitals and public areas [52]. Concerning the use of copper to control the spread of viruses such as SARS-CoV-2, Hewawaduge et al. (2021) reported that the viral load in CuS-impregnated masks was significantly reduced in in vitro assays [154]. These copper-based applications could be vital in reducing the spread of SARS-CoV-2 or other viruses.

In addition, it is necessary to understand how the use of copper surfaces and NPs affects microbial communities. For example, the effect of masks or clothing with copper fibers or the effect of NPs on the skin microbiota should be determined. On the other hand, due to their use in hospitals, it is relevant to determine what changes are produced by copper surfaces among the abundance of pathogens associated with HAIs. This point is appropriate considering that this metal could generate selection pressure among the organisms present in the surface community. In turn, it will be necessary to find elements that could enhance the antimicrobial effects of copper to make it more effective and specific to defined microbial genera/species, especially in the future, when new antimicrobial agents will be required in the face of multidrug-resistant organisms.

Another relevant point to consider is the effect of copper on human health and the environment. Copper NPs can be toxic in different organisms when used in inappropriate concentrations, causing inflammation in the pulmonary epithelium and accumulating in organs such as liver and kidney [155,156,157]. The indiscriminate and unregulated use of copper nanoparticles can be harmful to the environment, since the waste associated with the research and application of copper nanoparticle-related products ends up in landfills without any management [84,158]. Copper-rich wastes can pollute soils and water, negatively affecting the ecosystem. It is important for future research to address these limitations and to understand the mechanisms of copper toxicity, and then use that knowledge to improve NP properties and their interaction with the environment.

After analyzing the literature regarding Cu-based NPs, one limitation is observed that is related to the composition of the nanoparticles used in many studies. Fifty percent of the articles cited in Table 2 describe the use of nanoparticles as antimicrobials. However, 64.7% of these articles do not indicate the composition of the nanoparticles used (CuxS, CuO, CuxSx, or others), mainly due to problems associated with their characterization. This limitation affects the standardization and comparison between the cited articles. In future research, it will be essential to consider the characterization of nanoparticles in terms of composition, size, solubility, and stability. In turn, the methods used to determine the antimicrobial properties of copper NPs differ among the studies cited in Table 2. Inhibition halos, viable cell counts, DNA degradation studies, ROS production, lipid peroxidation, and membrane potential determination have been used [30,96]. In the case of viruses, different methodologies are regularly used to study the effect of NPs. Most studies evaluate the infective capacity of the virus in cell cultures exposed to copper-based nanoparticles; however, other studies evaluate the effect of NPs by mixing the viral particles, antigens, or proteins with the nanomaterials [15,47,73,125,128,131,133,134].

In conclusion, despite the enormous advance of the last few decades in understanding the effect of Cu surfaces and NPs on microorganisms, many unanswered questions about their antimicrobial effect remain. A total understanding of this process is essential, particularly considering the wide use of copper as an antimicrobial in different applications and the urgent necessity for developing new alternatives to deal with multidrug-resistant microorganisms.

## Data Availability

Not applicable.
